# Association between the preoperative C-reactive protein-albumin-lymphocyte index and prognosis after hepatic resection for hepatocellular carcinoma

**DOI:** 10.1371/journal.pone.0358776

**Published:** 2026-09-30

**Authors:** Tomonari Shimagaki, Atsushi Maeda, Tomoya Harima, Akihiro Sakai, Rumi Matono, Naotaka Hashimoto, Shohei Yamaguchi, Kozo Konishi, Kenkichi Hashimoto, Takashi Maeda

**Affiliations:** Department of Surgery, Hiroshima Red Cross Hospital and Atomic-bomb Survivors Hospital; Tottori University Faculty of Medicine Graduate School of Medicine: Tottori Daigaku Igakubu Daigakuin Igakukei Kenkyuka, JAPAN

## Abstract

**Background:**

Postoperative recurrence after hepatic resection for hepatocellular carcinoma (HCC) remains frequent. Readily available biomarkers that integrate host inflammatory, immune, and nutritional statuses may improve risk stratification. In this study, we evaluated the prognostic significance of the preoperative C-reactive protein (CRP)-albumin-lymphocyte (CALLY) index in patients who underwent resection for primary HCC.

**Methods:**

This retrospective, single-center study included 611 patients who underwent hepatic resection for primary HCC between 2004 and 2022. The CALLY index was defined as (albumin × lymphocytes)/(CRP × 10^4^). Patients were classified into high (≥ 2.2) and low (< 2.2) CALLY index groups. Clinicopathological factors, disease-free survival (DFS), and overall survival (OS) were compared. Prognostic factors were assessed using Cox proportional hazards models, and propensity-score matching was performed.

**Results:**

The low CALLY index group had lower albumin levels, higher CRP and alpha-fetoprotein levels, larger tumors, and more severe postoperative complications than the high CALLY index group. Patients with low CALLY index had significantly worse DFS and OS than those with high CALLY index. Multivariate analysis identified that the low CALLY index was independently associated with worse DFS (hazard ratio (HR), 1.86; 95% confidence interval (CI), 1.40–2.47; P < 0.0001) and OS (HR, 1.93; 95% CI, 1.37–2.72; P = 0.0002). After propensity-score matching, the low CALLY index group continued to have significantly poorer DFS and OS rates than the high CALLY index group.

**Conclusions:**

The preoperative CALLY index was independently associated with DFS and OS after hepatic resection for primary HCC. This simple biomarker may help identify patients at a high risk of recurrence and poor survival.

## Introduction

Hepatocellular carcinoma (HCC) is a leading cause of cancer-related mortality worldwide. Hepatic resection remains a potentially curative treatment for select patients with preserved liver function and technically resectable disease [1]. However, postoperative recurrence is common, even after resection with curative intent, and is a major determinant of long-term survival [2,3]. Therefore, accurate perioperative risk stratification is clinically important for tailoring surveillance intensity, selecting candidates for adjuvant or clinical trial strategies, and counseling patients on prognosis [3–5].

Prognosis after HCC resection is determined not only by tumor burden and pathological aggressiveness, but also by host-related factors, including hepatic functional reserve, systemic inflammation, nutritional status, and antitumor immunity [4,6,7]. Conventional liver function assessments, such as the Child-Pugh classification [6], model for end-stage liver disease (MELD) score, fibrosis-4 (FIB-4) index, indocyanine green retention rate at 15 min (ICG R15), and albumin–bilirubin (ALBI) grade [8] are widely used to guide treatment selection and estimate prognosis [9]. However, these measures do not fully capture the systemic inflammatory and immunonutritional milieu, which may influence recurrence and survival.

The C-reactive protein (CRP)-albumin-lymphocyte (CALLY) index is a simple composite marker that is calculated using routinely available laboratory parameters [10]. Because CRP reflects systemic inflammation, albumin reflects hepatic synthetic function and nutritional status, and lymphocyte count reflects host immune competence, the CALLY index may provide an integrated assessment of the biological conditions that influence postoperative outcomes [11,12]. Previous studies have suggested the prognostic utility of the CALLY index in patients with HCC and other gastrointestinal malignancies. However, further validation in independent surgical cohorts remains warranted [13,14].

In this study, we aimed to evaluate the prognostic significance of the preoperative CALLY index in patients who underwent hepatic resection for primary HCC. We compared the clinicopathological features and long-term outcomes between patients with high and low CALLY index values, examined the independent prognostic value of the CALLY index for disease-free survival (DFS) and overall survival (OS), compared its prognostic discrimination with established liver function indices, and performed propensity-score matching to reduce baseline imbalance between the groups.

## Materials and methods

### Study design

In this retrospective cohort study, we reviewed 1089 consecutive patients who underwent hepatic resection for liver tumors at Hiroshima Red Cross Hospital and Atomic-bomb Survivors Hospital between 2004 and 2022. Among 1,089 consecutive patients who underwent hepatic resection for liver tumors between 2004 and 2022, 478 patients were excluded for the following reasons: non-HCC liver tumors (n = 68), recurrent HCC (n = 381), and missing laboratory data required for calculation of the CALLY index (n = 29). Finally, 611 patients who underwent initial hepatic resection for primary HCC were included in the analysis. The detailed patient selection process is shown in Fig 1. This retrospective study was conducted in accordance with the Declaration of Helsinki and was approved by the Ethics and Indications Committee of Hiroshima Red Cross Hospital and Atomic-bomb Survivors Hospital (approval No. 2021−029). Data were accessed for research purposes on June 24, 2025. Because of the retrospective observational design, the requirement for written informed consent was waived by the ethics committee. Information about the study was disclosed through an opt-out approach, and patients or their representatives were given the opportunity to decline participation. For patients who had died or could not be contacted, the opt-out procedure approved by the ethics committee was applied. Authorized investigators accessed identifiable clinical records only for data collection. After data extraction, direct identifiers were removed, and statistical analyses were performed using a de-identified dataset.

**Fig 1:**
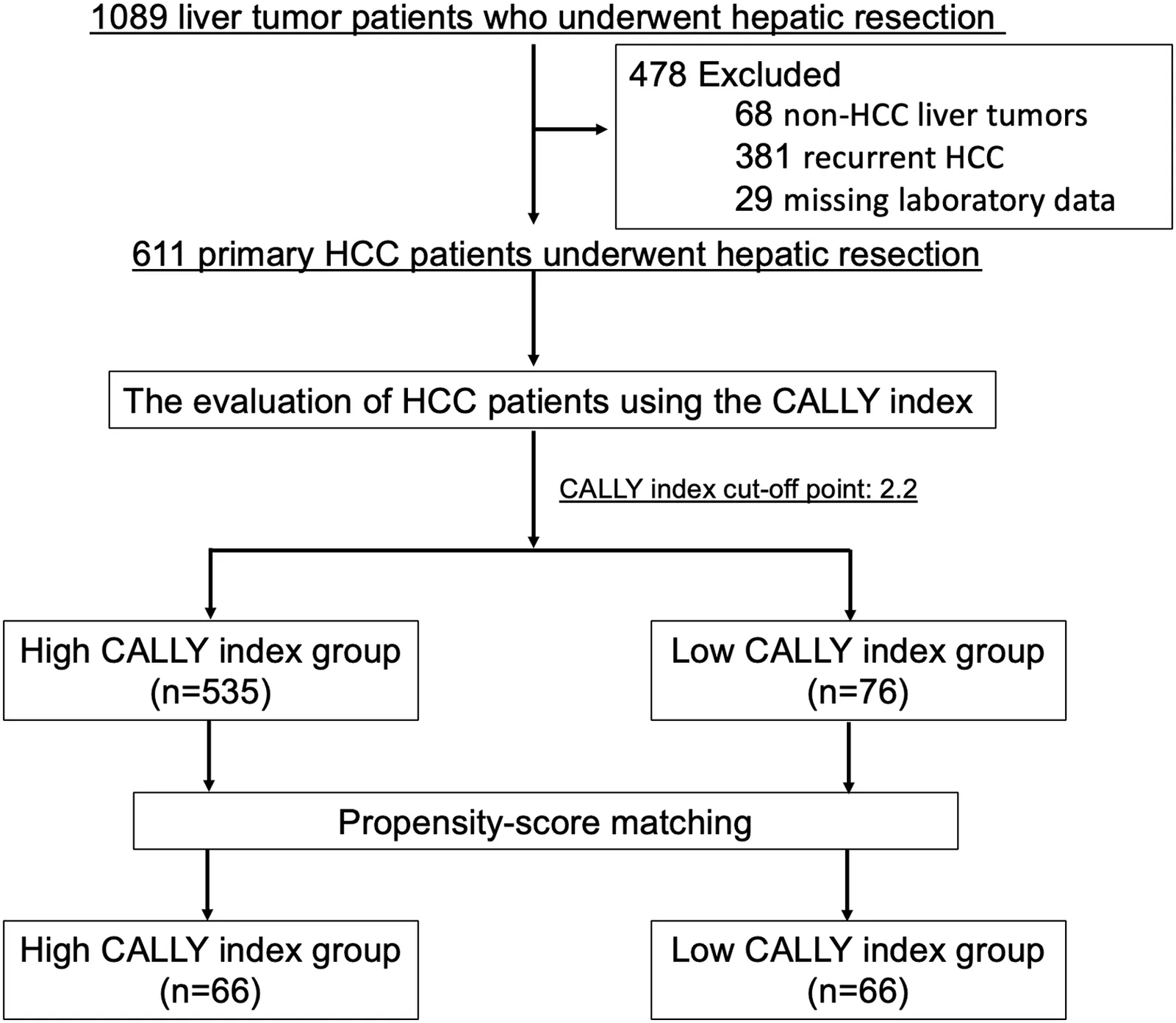
Study flow diagram. Among 1,089 patients who underwent hepatic resection for liver tumors between 2004 and 2022, 478 were excluded because of non-HCC liver tumors (n = 68), recurrent HCC (n = 381), or missing laboratory data required for calculation of the CALLY index (n = 29). Finally, 611 patients who underwent initial hepatic resection for primary HCC were included. Patients were divided into the high CALLY index group (≥ 2.2; n = 535) and low CALLY index group (< 2.2; n = 76), and propensity-score matching generated 66 matched pairs.

All relevant data underlying the findings of this study are provided in the Supporting Information files. The de-identified minimal dataset used to reproduce the baseline tables, survival analyses, time-dependent ROC analyses, and propensity-score matching analysis is provided as S1 Dataset.

### Preoperative evaluation and treatment

Preoperative evaluations included laboratory tests, liver function assessment, and tumor marker and imaging studies. Hepatic functional reserve was assessed using standard clinical parameters, including serum albumin, bilirubin, prothrombin time, platelet count, ICG R15, ALBI score, easy-ALBI (EZ-ALBI) score [15], Child-Pugh classification, MELD score, and FIB-4 index, as appropriate [8]. The extent of hepatic resection and operative approach were determined according to the tumor location and extent, liver functional reserve, and general condition of the patient. Open and laparoscopic resections were performed.

### Definition of the CALLY index

The preoperative CALLY index was calculated using laboratory values obtained before surgery according to the following formula: CALLY index = (serum albumin (g/dL) × lymphocyte count (cells/μL))/(CRP (mg/dL) × 10^4^) [10]. The cutoff value of the CALLY index was determined using receiver operating characteristic curve analysis with overall survival as the outcome. The optimal cutoff value was selected using the Youden index, and the resulting cutoff value was 2.2. The ROC curve used for cutoff selection is shown in Supplementary Figure 1. Because this cutoff was derived from the present cohort and was not prespecified from an external dataset, it was considered exploratory. To reduce the limitations associated with dichotomization, the CALLY index was also analyzed as a continuous variable in Cox proportional hazards models.

### Laboratory measurements and calculation of the CALLY index

Preoperative laboratory data were obtained within 7 days before hepatic resection. The CALLY index was calculated using serum albumin, peripheral lymphocyte count, and CRP values measured before surgery. Serum albumin was expressed in g/dL, lymphocyte count in cells/μL, and CRP in mg/dL. Patients with clinically apparent active infection or acute inflammatory disease at the time of preoperative blood sampling were excluded where identified from the medical records. The lower reporting limit for CRP at our institution was 0.01 mg/dL. No patient had a CRP value of zero. Therefore, all CALLY index values could be calculated directly.

### Outcomes and follow-up

The primary outcomes were OS and DFS after hepatic resection. OS was defined as the interval from the date of surgery to death from any cause or last follow-up. DFS was defined as the interval between the date of surgery and the first documented recurrence or death. Early recurrence was defined as recurrence within 1 year after surgery. Postoperative surveillance was performed using tumor marker assessment and imaging examinations according to institutional practice. The patients were followed up every 3–6 months until death or loss to follow-up. Follow-up duration was calculated from the date of surgery to the date of last follow-up or death.

### Sensitivity analysis according to treatment era

Because the study period extended from 2004 to 2022, temporal changes in surgical techniques, imaging modalities, surveillance strategies, antiviral treatment, and treatment after recurrence may have influenced postoperative outcomes. Therefore, we performed a sensitivity analysis according to treatment era. Patients were divided into two groups according to the year of surgery: 2004–2013 and 2014–2022. OS and DFS were compared between the high and low CALLY index groups within each treatment era using the Kaplan–Meier method and log-rank test.

### Treatment after recurrence

Treatment after recurrence was reviewed from the medical records and classified according to the initial treatment administered for the first recurrence. Treatment categories included repeat hepatic resection, radiofrequency ablation, transarterial chemoembolization or transarterial embolization, systemic therapy, radiotherapy, best supportive care, and unknown or unavailable treatment information.

### Clinicopathological variables

Clinicopathological variables included sex, age, hypertension, diabetes mellitus, viral hepatitis status, liver function parameters, inflammatory markers, tumor markers such as alpha-fetoprotein (AFP) and des-γ-carboxy prothrombin (DCP), maximum tumor size, tumor number, operative variables, postoperative complications, vascular invasion, tumor differentiation, and intrahepatic metastasis. Postoperative complications were graded according to the Clavien–Dindo classification, and grade III or higher complications were considered severe.

Variables were selected for multivariable Cox models based on clinical relevance and statistical significance in univariate analysis. To avoid model overfitting, the number of covariates was considered in relation to the number of observed events. Variables that were components of the CALLY index were not entered into the same model as the CALLY index. In addition, ALBI and EZ-ALBI scores include albumin-related information; therefore, models including the CALLY index and albumin-based liver function indices were interpreted with caution because of potential collinearity.

Missing data were observed for diabetes mellitus in one patient. Complete-case analysis was performed for variables included in each model.

### Propensity score matching analysis

Propensity-score matching was performed as a sensitivity analysis to reduce measured baseline imbalance between patients with high and low CALLY index values. The propensity score was estimated using a logistic regression model in which the dependent variable was the CALLY index group. The covariates included liver function, tumor multiplicity, viral etiology, AFP level, DCP level, maximum tumor size, and vascular invasion. These variables were selected based on clinical relevance and established prognostic importance after hepatic resection for HCC. The individual components of the CALLY index, including albumin, CRP, and lymphocyte count, were not included in the propensity-score model to avoid overadjustment.

One-to-one nearest-neighbor matching was performed without replacement using a caliper width of 0.2 of the standard deviation of the logit of the propensity score. Patients outside the region of common support were not matched. Complete-case analysis was performed for the variables included in the propensity-score model. Covariate balance before and after matching was assessed using standardized mean differences, and an absolute standardized mean difference <0.1 was considered to indicate adequate balance. Survival outcomes in the matched cohort were evaluated using Kaplan–Meier curves and compared using the log-rank test. Because the CALLY index is a prognostic biomarker rather than a treatment exposure, the matched analysis was interpreted as a sensitivity analysis of prognostic association rather than a causal effect analysis.

### Statistical analysis

Continuous variables are presented as median (interquartile range), and categorical variables are presented as numbers and percentages. Differences between the groups (high vs. low CALLY index group) were assessed using the Mann–Whitney U test. Associations between variables were determined using Fisher’s exact test or the chi-square test, as appropriate. Survival curves were estimated using the Kaplan–Meier method and compared using the log-rank test. Cox proportional hazards regression models were used to identify factors associated with DFS and OS. The proportional hazards assumption was assessed graphically using survival plots and residual diagnostics available. Variables with clinical relevance or statistical significance in the univariate analysis were used in multivariate models. Some continuous variables were categorized using clinically relevant or cohort-derived thresholds for Cox regression analyses; however, the CALLY index was additionally analyzed as a continuous variable in sensitivity analyses. Receiver operating characteristic (ROC) curves were used to compare the prognostic discrimination of the CALLY index with the established liver function indices for predicting DFS, early recurrence, and OS. Statistical analyses were performed using JMP Pro software (version 19.0.1; SAS Institute, Cary, NC, USA). Statistical significance was set at P < 0.05.

Because DFS and OS are censored time-to-event outcomes, conventional ROC analysis was not used as the primary method for evaluating prognostic discrimination for these endpoints. Time-dependent ROC analyses were performed using EZR (version 1.70; Jichi Medical University, Tochigi, Japan), which is a graphical user interface for R (The R Foundation for Statistical Computing, Vienna, Austria) at prespecified time points of 1, 3, and 5 years after hepatic resection. Time-dependent AUCs were calculated for the CALLY index and established liver function indices, including ALBI score, EZ-ALBI score, FIB-4 index, MELD score, and Child-Pugh classification. Conventional ROC analysis was retained only for early recurrence within 1 year after surgery, which was evaluated as a binary outcome.

## Results

### Patient characteristics

The flowchart of this study is shown in Fig 1. Of the 1,089 patients who underwent hepatic resection for liver tumors during the study period, 478 were excluded: 68 had non-HCC liver tumors, 381 had recurrent HCC, and 29 had missing laboratory data required for calculation of the CALLY index. The final cohort consisted of 611 patients with primary HCC. The cohort included 422 men and 189 women, with a median age of 71 years (interquartile range, 64–77 years) (Table 1). The underlying liver diseases included hepatitis B virus infection (101 patients), hepatitis C virus infection (349 patients), and non-B, non-C disease (161 patients). Based on a preoperative CALLY index cutoff of 2.2, 535 patients were classified into the high CALLY index group and 76 into the low CALLY index group. During follow-up, DFS events occurred in 369 patients and 242 patients were censored for DFS. For OS, death occurred in 233 patients and 378 patients were censored.

**Table 1:** Comparative analysis of clinicopathological parameters between the high and low CALLY index groups.

Variable	Total (n = 611)	High CALLY index group (n = 535)	Low CALLY index group (n = 76)	*P-value*
Sex (male/female)	422/189	368/167	54/22	0.6890
Age (years)	71 (64–77)	71 (64–77)	71 (62–77)	0.5767
Hypertension (yes/no)	349/262	302/233	47/29	0.3834
DM (yes/no)	225/385	194/340	31/45	0.4509
HBV/HCV/NBNC	101/349/161	89/317/129	12/32/32	**0.0030**
Serum albumin (g/dL)	4.1 (3.7-4.3)	4.1 (3.7-4.4)	3.8 (3.5-4.1)	**< 0.0001**
Total bilirubin (mg/dL)	0.7 (0.6-1.0)	0.7 (0.6-0.9)	0.8 (0.5-1.1)	0.1939
AST (IU/L)	36 (25-52)	35 (25-52)	41 (28-57)	0.5055
ALT (IU/L)	33 (23-51)	33 (23-51)	35 (21-52)	0.9367
PT (%)	92 (83-101)	92 (83-101)	90 (78-97)	0.1791
Platelet (×10^4^/μL)	14.4 (10.1-18.1)	13.9 (10.0-17.7)	16.6 (10.5-21.9)	**0.0043**
ICG R15 (%)	13.9 (9.0-21.7)	13.7 (8.9-21.3)	15.3 (9.8-22.6)	0.2085
CRP (mg/dL)	0.06 (0.05-0.16)	0.05 (0.05-0.11)	0.62 (0.40-1.90)	**< 0.0001**
ALBI score	−2.7 (−3.0--2.4)	−2.7 (−3.0--2.5)	−2.5 (−2.8--2.2)	**< 0.0001**
ALBI grade, 1/2/3	380/231/0	352/183/0	28/48/0	**< 0.0001**
EZ-ALBI score	−35.6 (−38.2--32.7)	−36.1 (−38.4--32.8)	−32.8 (−36.1--30.1)	**< 0.0001**
EZ-ALBI grade, 1/2/3	379/232/0	350/185/0	29/47/0	**< 0.0001**
AFP (ng/mL)	11.1 (4.5–47.0)	10.9 (4.4–47.0)	12.7 (4.9–61.0)	**0.0005**
DCP (mAU/mL)	50 (24–172)	48 (23–160)	106 (26–892)	**< 0.0001**
Maximum tumor size (cm)	2.4 (1.7-3.5)	2.3 (1.7-3.3)	3.4 (1.8-5.9)	**< 0.0001**
Tumor nodules (single/multiple)	445/166	390/145	55/21	0.9227
Operation time (min)	222 (172-294)	220 (172-293)	246 (169-311)	0.0585
Blood loss (mL)	270 (115-553)	265 (118-557)	311 (87-533)	0.9568
Open surgery/laparoscopic surgery	560/51	494/41	66/10	0.1051
Postoperative complication CD (0–2/ ≥ 3)	561/50	496/39	65/11	**0.0325**
Vascular invasion (vp, vv, va) (yes/no)	227/384	197/338	30/46	0.6545
Differentiation (well/moderate/poor)	46/413/152	42/367/126	4/46/26	0.1181
IM (yes/no)	85/526	69/466	16/60	0.0546

CALLY, C-reactive protein-albumin-lymphocyte; DM, diabetes mellitus; HBV, hepatitis B virus; HCV, hepatitis C virus; NBNC, non-B non-C hepatitis; AST, aspartate aminotransferase; ALT, alanine aminotransferase; PT, prothrombin time; ICG R15, indocyanine green retention rate at 15 min; ALBI score, Albumin-Bilirubin score; EZ-ALBI score, Easy Albumin-Bilirubin score; AFP, alpha-fetoprotein; DCP, des-γ-carboxy prothrombin; CD, Clavien–Dindo classification; vp, portal vein invasion; vv, hepatic vein invasion; va, hepatic artery invasion; IM, intrahepatic metastasis

Continuous variables are presented as median (interquartile range), and categorical variables are presented as numbers. P values were calculated using the Mann–Whitney U test, chi-square test, or Fisher’s exact test, as appropriate.

Boldface p-values indicate statistical significance.

Data were missing for diabetes mellitus in one patient. The number of patients with available data is shown where applicable.

Missing data were minimal. Diabetes mellitus status was missing in one patient, whereas data for albumin, CRP, lymphocyte count, AFP, DCP, tumor size, vascular invasion, and survival outcomes were available for all 611 patients. The details of missing data are shown in Supplementary Table 1.

### Treatment after recurrence

Among patients who developed recurrence, treatment after recurrence varied according to recurrence pattern, hepatic functional reserve, general condition, and treatment era. The initial treatment for recurrence included repeat hepatic resection, radiofrequency ablation, transarterial chemoembolization or transarterial embolization, systemic therapy, radiotherapy, and best supportive care. The distribution of initial post-recurrence treatment is shown in Supplementary Table 2.

### Comparison of clinicopathological characteristics according to the CALLY index

The clinicopathological characteristics are summarized in Table 1. Compared with the high CALLY index group, the low CALLY index group had significantly lower serum albumin levels (P < 0.0001), higher CRP levels (P < 0.0001), higher platelet counts (P = 0.0043), worse ALBI and EZ-ALBI scores, higher AFP levels, and larger tumors (P < 0.0001). Severe postoperative complications were also more frequent in the low CALLY index group (P = 0.0325). The high and low CALLY index groups showed no significant differences in terms of sex, age, diabetes mellitus, total bilirubin, transaminases, ICG R15, operative blood loss, tumor multiplicity, vascular invasion, differentiation, or intrahepatic metastasis.

### Poor DFS and OS in the low CALLY index group

The low CALLY index group had significantly worse OS and DFS than the high CALLY index group (both, P < 0.0001) (Fig 2A, B). These results indicated that the low CALLY index group had a high HCC recurrence rate and poor prognosis.

**Fig 2:**
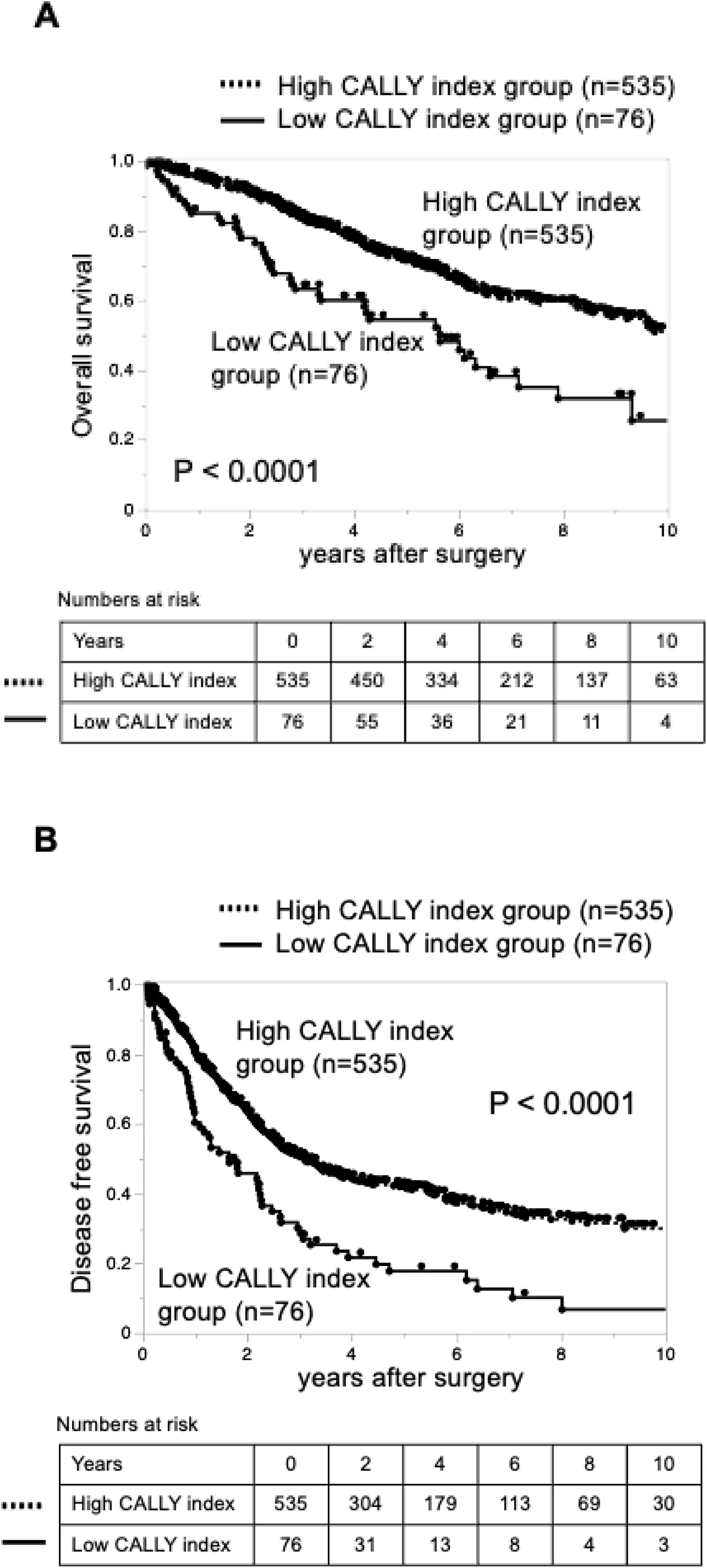
Kaplan–Meier survival curves according to the preoperative CALLY index. (A) Overall survival. (B) Disease-free survival. P values were calculated using the log-rank test. Tick marks indicate censored observations.

### Sensitivity analysis by treatment era

To evaluate the possible influence of temporal changes in treatment and surveillance, survival analyses were performed separately according to treatment era. In the 2004–2013 cohort, the low CALLY index group had significantly worse OS and DFS than the high CALLY index group (OS, P = 0.0003; DFS, P = 0.0012). Similarly, in the 2014–2022 cohort, the low CALLY index group had significantly worse OS and DFS than the high CALLY index group (OS, P = 0.0024; DFS, P = 0.0002). These findings suggest that the association between low CALLY index and poor postoperative outcomes was consistent across treatment eras (Supplementary Figure 2).

### Prognostic factors for DFS

Univariate Cox regression analysis identified several factors associated with DFS, including serum albumin, aspartate aminotransferase (AST), alanine aminotransferase, ICG R15, AFP, DCP, maximum tumor size, tumor multiplicity, vascular invasion, poor differentiation, intrahepatic metastasis, ALBI grade, EZ-ALBI grade, and CALLY index. In the multivariate analysis, AFP, DCP, tumor multiplicity, intrahepatic metastasis, and a low CALLY index were independently associated with DFS. A low CALLY index was associated with an approximately 1.9-fold higher risk of recurrence (hazard ratio [HR], 1.86; 95% confidence interval [CI], 1.40–2.47; P < 0.0001; Table 2).

**Table 2:** Univariate and multivariate Cox regression analyses of the clinicopathological variables related to disease-free survival after hepatic resection for hepatocellular carcinoma.

Variable	Univariate analysis			Multivariate analysis		
	HR	95% CI	*P-value*	HR	95% CI	*P-value*
Sex (male)	1.05	0.85–1.32	0.6228			
Age (≥ 69.8 vs. < 69.8)	1.20	0.97–1.47	0.0871			
Hypertension (yes/no)	1.03	0.84–1.26	0.7947			
DM (yes/no)	0.94	0.76–1.16	0.5415			
Serum albumin (≦ 3.5 vs. > 3.5)	1.52	1.10–2.10	**0.0116**			
Total bilirubin (≦ 0.8 vs. > 0.8)	0.92	0.75–1.13	0.4404			
Platelet (≦ 15.1 vs. > 15.1 × 10^4^)	1.23	0.84–1.49	0.1816			
AST (≦ 44 vs. > 44)	0.80	0.65–0.98	**0.0335**	0.89	0.64–1.22	0.4599
ALT (≦ 42 vs. > 42)	0.81	0.66–0.99	**0.0485**	0.96	0.69–1.33	0.7971
ICG R15 (≦ 16.4 vs. > 16.4%)	0.79	0.64–0.97	**0.0249**	0.84	0.68–1.04	0.1181
AFP (≦ 11.1 vs. > 11.1)	0.65	0.53–0.80	**< 0.0001**	0.78	0.63–0.97	**0.0280**
DCP (≦ 50 vs. > 50)	0.59	0.48–0.72	**< 0.0001**	0.70	0.56–0.87	**0.0011**
Maximum tumor size (≦ 3.1 vs. > 3.1 cm)	0.74	0.59–0.92	**0.0064**	0.84	0.67–1.06	0.1368
Tumor nodules (single/multiple)	0.54	0.44–0.68	**< 0.0001**	0.64	0.51–0.81	**0.0002**
Open surgery/laparoscopic surgery	0.78	0.53–1.15	0.2086			
Postoperative complication CD (0–2/ ≥ 3)	0.80	0.54–1.19	0.2694			
Vascular invasion (vp, vv, va) (yes/no)	1.38	1.12–1.70	**0.0024**	1.15	0.91–1.44	0.2392
Differentiation (poorly)	1.34	1.06–1.68	**0.0144**	1.09	0.86–1.40	0.4713
IM (yes/no)	2.06	1.55–2.72	**< 0.0001**	1.46	1.08–1.97	**0.0126**
ALBI grade 1/2	0.71	0.58–0.87	**0.0011**			
EZ-ALBI grade 1/2	0.68	0.55–0.83	**0.0002**			
CALLY index (< 2.2 vs. ≥ 2.2)	1.94	1.47–2.57	**< 0.0001**	1.86	1.40–2.47	**< 0.0001**
CALLY index, continuous	0.98	0.97-0.99	**< 0.0001**			

HR, hazard ratio; CI, confidence interval; DM, diabetes mellitus; AST, aspartate aminotransferase; ALT, alanine aminotransferase; ICG R15, indocyanine green retention rate at 15 min; AFP, alpha-fetoprotein; DCP, des-γ-carboxy prothrombin; CD, Clavien–Dindo classification; vp, portal vein invasion; vv, hepatic vein invasion; va, hepatic artery invasion; IM, intrahepatic metastasis; ALBI, Albumin-Bilirubin; EZ-ALBI, Easy Albumin-Bilirubin; CALLY, C-reactive protein-albumin-lymphocyte

Bold p-values are statistically significant

### Prognostic factors for OS

Univariate Cox regression analysis showed that age, serum albumin, AST, ICG R15, AFP, DCP, maximum tumor size, tumor multiplicity, severe postoperative complications, vascular invasion, poor differentiation, intrahepatic metastasis, ALBI grade, EZ-ALBI grade, and CALLY index were associated with OS. In multivariate analysis, age, ICG R15, AFP, DCP, maximum tumor size, tumor multiplicity, severe postoperative complications, poor differentiation, intrahepatic metastasis, and low CALLY index remained independently associated with OS. A low CALLY index was independently associated with worse OS (HR, 1.93; 95% CI, 1.37–2.72; P = 0.0002; Table 3).

**Table 3:** Univariate and multivariate Cox regression analyses of the clinicopathological variables related to overall survival after hepatic resection for hepatocellular carcinoma.

Variable	Univariate analysis			Multivariate analysis		
	HR	95% CI	*P-value*	HR	95% CI	*P-value*
Sex (male)	1.17	0.89–1.54	0.2696			
Age (≥ 69.8 vs. < 69.8)	1.68	1.29–2.20	**0.0002**	1.71	1.30–2.25	**0.0001**
Hypertension (yes/no)	1.05	0.81–1.36	0.7221			
DM (yes/no)	1.01	0.78–1.32	0.9227			
Serum albumin (≦ 3.5 vs. > 3.5)	1.98	1.37–2.87	**0.0003**			
Total bilirubin (≦ 0.8 vs. > 0.8)	0.82	0.64–1.06	0.1356			
Platelet (≦ 15.1 vs. > 15.1 × 10^4^)	1.26	0.79–1.46	0.2582			
AST (≦ 44 vs. > 44)	0.73	0.56–0.94	**0.0169**	0.88	0.67–1.15	0.3516
ALT (≦ 42 vs. > 42)	0.95	0.73–1.24	0.7167			
ICG R15 (≦ 16.4 vs. > 16.4%)	0.59	0.45–0.76	**< 0.0001**	0.64	0.49–0.84	**0.0012**
AFP (≦ 11.1 vs. > 11.1)	0.49	0.37–0.64	**< 0.0001**	0.62	0.46–0.82	**0.0009**
DCP (≦ 50 vs. > 50)	0.50	0.39–0.66	**< 0.0001**	0.68	0.51–0.89	**0.0062**
Maximum tumor size (≦ 3.1 vs. > 3.1 cm)	0.60	0.46–0.79	**0.0002**	0.75	0.56–0.99	**0.0489**
Tumor nodules (single/multiple)	0.61	0.47–0.81	**0.0004**	0.75	0.56–0.99	**0.0475**
Open surgery/laparoscopic surgery	0.83	0.43–1.63	0.5970			
Postoperative complication CD (0–2/ ≥ 3)	0.42	0.28–0.62	**< 0.0001**	0.61	0.40–0.92	**0.0180**
Vascular invasion (vp, vv, va) (yes/no)	1.55	1.19–2.00	**0.0010**	1.03	0.77–1.37	0.8358
Differentiation (poorly)	1.94	1.48–2.55	**< 0.0001**	1.53	1.14–2.07	**0.0051**
IM (yes/no)	2.66	1.94–3.66	**< 0.0001**	1.90	1.35–2.67	**0.0002**
ALBI grade 1/2	0.49	0.38–0.63	**< 0.0001**			
EZ-ALBI grade 1/2	0.48	0.37–0.62	**< 0.0001**			
CALLY index (< 2.2 vs. ≥ 2.2)	2.10	1.50–2.93	**< 0.0001**	1.93	1.37–2.72	**0.0002**
CALLY index, continuous	0.97	0.96-0.98	**< 0.0001**			

HR, hazard ratio; CI, confidence interval; DM, diabetes mellitus; AST, aspartate aminotransferase; ALT, alanine aminotransferase; ICG R15, indocyanine green retention rate at 15 min, AFP, alpha-fetoprotein; DCP, des-γ-carboxy prothrombin; CD, Clavien–Dindo classification; vp, portal vein invasion; vv, hepatic vein invasion; va, hepatic artery invasion; IM, intrahepatic metastasis; ALBI, Albumin-Bilirubin; EZ-ALBI, Easy Albumin-Bilirubin; CALLY, C-reactive protein-albumin-lymphocyte.

Bold p-values are statistically significant.

In addition to the cutoff-based analysis, the CALLY index was analyzed as a continuous variable. Lower CALLY index values were associated with poorer DFS and OS in Cox proportional hazards models (Table 2 and 3).

### Prognostic discrimination of the CALLY index

Because DFS and OS were censored time-to-event outcomes, prognostic discrimination was evaluated using time-dependent ROC analysis. The time-dependent AUCs of the CALLY index for DFS at 1, 3, and 5 years were 0.523, 0.529, and 0.509, respectively. The corresponding AUCs for OS were 0.543, 0.541, and 0.548, respectively (Table 4). The CALLY index showed numerically favorable discrimination for OS (Table 4) and early recurrence within 1 year after surgery (Table 5) compared with several liver function indices; however, the absolute AUC was modest. Overall, the CALLY index showed modest prognostic discrimination. Therefore, the results suggest that the CALLY index may provide adjunctive prognostic information rather than serving as a stand-alone predictor.

**Table 4:** Time-dependent area under the curve (AUC) for disease-free survival (DFS) and overall survival (OS).

Index	1-year DFS AUC	3-year DFS AUC	5-year DFS AUC	1-year OS AUC	3-year OS AUC	5-year OS AUC
CALLY index	0.523	0.529	0.509	0.543	0.541	0.548
ALBI score	0.569	0.544	0.570	0.444	0.474	0.508
EZ-ALBI score	0.564	0.550	0.566	0.449	0.486	0.500
Child-Pugh classification	0.541	0.527	0.508	0.486	0.476	0.487
MELD score	0.507	0.538	0.550	0.400	0.506	0.539
FIB-4 index	0.636	0.636	0.616	0.530	0.508	0.502

AUC, area under the curve; DFS, disease-free survival; OS, overall survival; CALLY, C-reactive protein-albumin-lymphocyte; ALBI, Albumin-Bilirubin; EZ-ALBI, Easy Albumin-Bilirubin; MELD, model for end-stage liver disease; FIB-4, fibrosis-4.

**Table 5:** Diagnostic accuracy of liver function assessment for predicting early recurrence (within 1 year after surgery) of resected hepatocellular carcinoma (n = 611).

Index	AUC	Sensitivity (%)	Specificity (%)
CALLY index	0.60121	53.5	63.8
EZ-ALBI score	0.56061	91.3	18.6
ALBI score	0.56991	90.6	23.6
Child-Pugh classification	0.55823	29.1	83.1
MELD score	0.51324	81.9	25.6
FIB-4 index	0.54309	23.6	86.2

AUC, area under the curve; CALLY, C-reactive protein-albumin-lymphocyte; EZ-ALBI, Easy Albumin-Bilirubin; ALBI, Albumin-Bilirubin; MELD, model for end-stage liver disease; FIB-4, fibrosis-4.

### Propensity-score matched analysis

Propensity-score matching generated 66 matched pairs. After matching, baseline covariates were better balanced between the high and low CALLY index groups. The absolute standardized mean differences of the matched covariates were reduced after matching, and most variables showed an absolute standardized mean difference <0.1 (Table 6). In the matched cohort, Kaplan–Meier curves showed significant separation between the low and high CALLY index groups for OS (P = 0.0234; Fig 3A) and DFS (P = 0.0317; Fig 3B). Because the CALLY index is a prognostic biomarker rather than a treatment exposure, this matched analysis was interpreted as a sensitivity analysis of prognostic association.

**Table 6:** Patient characteristics in the high and low CALLY index groups after propensity-score matching.

Variable	High CALLY index group (n = 66)	Low CALLY index group (n = 66)	*P-value*
Sex (male/female)	51/15	49/17	0.6846
Age (years)	70 (62-77)	70 (62-78)	0.8197
DM (yes/no)	27/39	26/40	0.8591
HBV/HCV/NBNC	12/29/25	11/29/26	0.9689
Performance status (0–1/ ≥ 2)	66/0	65/1	0.3155
Total bilirubin (mg/dL)	0.7 (0.5-1.1)	0.7 (0.6-1.0)	0.6827
AST (IU/L)	39 (28-52)	36 (25-53)	0.4290
ALT (IU/L)	33 (21-52)	32 (24-50)	0.3790
PT (%)	90 (77-97)	95 (85-101)	0.3608
Platelet (×10^4^/μL)	16.6 (10.5-20.6)	16.8 (11.2-22.7)	0.3192
ICG R15 (%)	15.3 (10.5-22.3)	12.5 (10.0-19.6)	0.3108
AFP (ng/mL)	14.7 (4.3–77.0)	11.1 (4.4–28.9)	0.5641
DCP (mAU/mL)	60 (24–450)	64 (24–233)	0.5907
Maximum tumor size (cm)	3.0 (1.8-4.5)	3.3 (2.0-5.1)	0.3749
Tumor nodules (single/multiple)	48/18	49/17	0.8437
Operation time (min)	229 (164-297)	246 (175-320)	0.5301
Blood loss (mL)	284 (84-502)	263 (145-635)	0.5024
Postoperative complication CD (0–2/ ≥ 3)	63/3	58/8	0.1154
Vascular invasion (vp, vv, va) (yes/no)	31/35	26/40	0.3796
Differentiation (poorly)	18/48	20/46	0.7006
IM (yes/no)	8/58	12/54	0.3315

CALLY, C-reactive protein-albumin-lymphocyte; DM, diabetes mellitus; HBV, hepatitis B virus; HCV, hepatitis C virus; NBNC, non-B non-C hepatitis; AST, aspartate aminotransferase; ALT, alanine aminotransferase; PT, prothrombin time; ICG R15, indocyanine green retention rate at 15 min; AFP, alpha-fetoprotein; DCP, des-γ-carboxy prothrombin; CD, Clavien–Dindo classification; vp, portal vein invasion; vv, hepatic vein invasion; va, hepatic artery invasion; IM, intrahepatic metastasis

Continuous variables are presented as median (interquartile range), and categorical variables are presented as numbers. P values were calculated using the Mann–Whitney U test, chi-square test, or Fisher’s exact test, as appropriate.

Boldface p-values indicate statistical significance.

**Fig 3:**
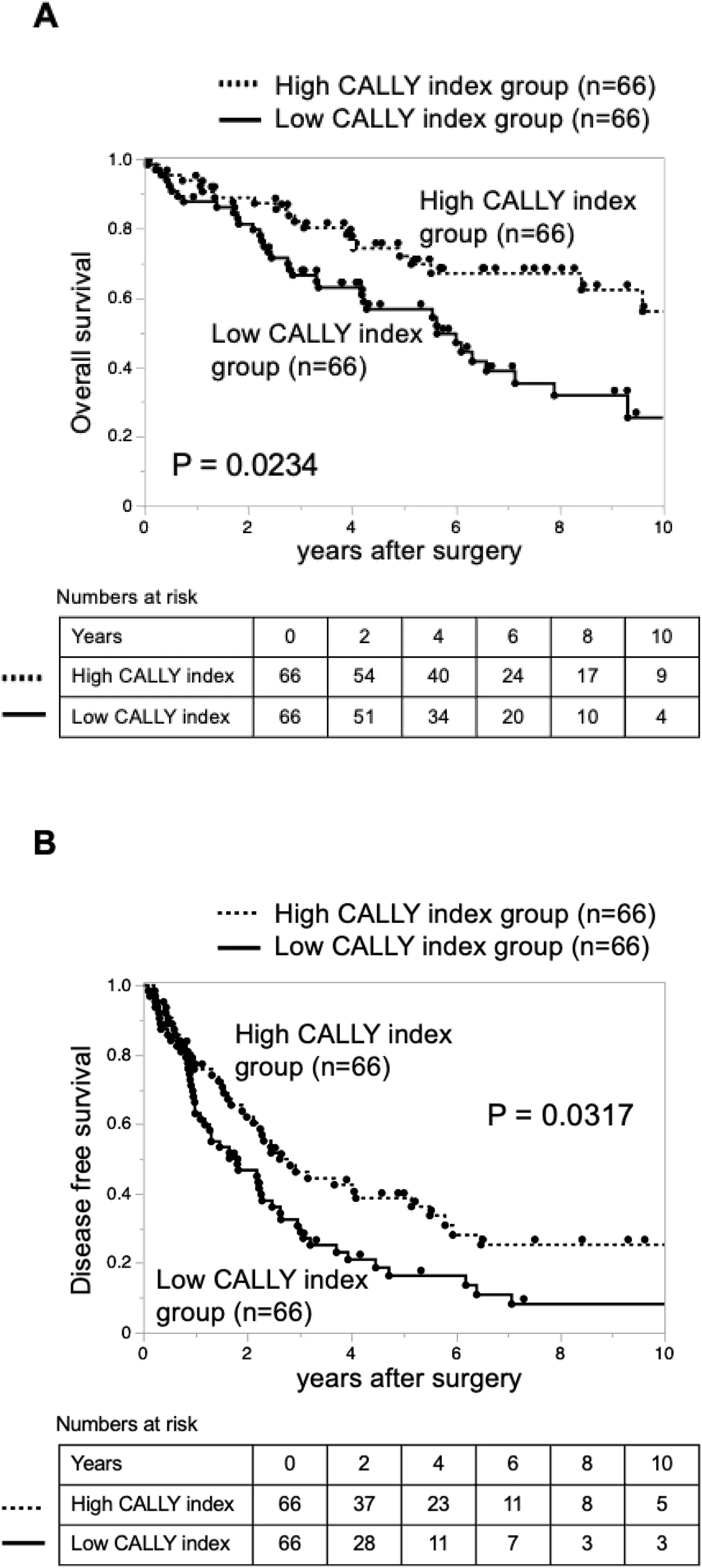
Kaplan–Meier survival curves according to the preoperative CALLY index after propensity-score matching. (A) Overall survival. (B) Disease-free survival. P values were calculated using the log-rank test. Tick marks indicate censored observations.

## Discussion

In this retrospective study that included 611 patients who underwent hepatic resection for primary HCC, the preoperative CALLY index was significantly associated with long-term outcomes. Patients with a low CALLY index had worse inflammatory and nutritional profiles, larger tumors, and poorer postoperative survival than those with high CALLY index. Importantly, a low CALLY index remained independently associated with both DFS and OS after adjusting for established tumor- and liver-related prognostic factors. The adverse prognostic association of a low CALLY index was confirmed after propensity-score matching, suggesting that this marker provides clinically relevant prognostic information beyond conventional clinicopathological variables.

The CALLY index has several advantages as a prognostic marker [13,16]. Elevated CRP is a marker of systemic inflammation that promotes tumor progression through cytokine-mediated tumor proliferation, angiogenesis, immune suppression, and cancer cachexia [12,17,18]. Albumin reflects the hepatic synthetic capacity and nutritional reserve, both of which are essential for tolerating major surgery and maintaining the antitumor defense [19–21]. Lymphocyte count reflects host cellular immunity, and lymphopenia may indicate impaired immune surveillance against residual micrometastatic disease [22]. By combining these three parameters, the CALLY index can capture the interactions between tumor-related inflammation, host immune competence, nutritional status, and hepatic functional reserve.

The present findings are consistent with those of previous reports showing that the CALLY index is associated with the prognosis after hepatectomy for HCC [10]. However, this study adds clinically useful information by evaluating a large single-center cohort of patients with primary HCC, comparing the CALLY index with multiple liver function indices, and confirming survival differences after propensity-score matching. The persistent prognostic separation after matching suggests that the CALLY index is not merely a surrogate for tumor size, liver function, or viral status but may represent an integrated host-response phenotype that influences postoperative recurrence and survival.

Risk stratification after HCC resection remains challenging because recurrence occurs through heterogeneous mechanisms, including intrahepatic metastasis from the primary tumor and de novo multicentric carcinogenesis in the diseased liver remnant [23,24]. Traditional prognostic models primarily focus on tumor burden, vascular invasion, and liver function [6]. The CALLY index may complement these parameters because it can be easily obtained from routine preoperative blood tests and does not require specialized assays [25]. Clinically, patients with low CALLY index may warrant closer postoperative surveillance, optimization of nutritional and inflammatory status, and consideration of adjuvant strategies or clinical trials, when available.

In the present analysis, we used time-dependent ROC analysis because DFS and OS are censored time-to-event outcomes. The CALLY index showed modest prognostic discrimination for OS. Therefore, the CALLY index should not be interpreted as a stand-alone prognostic tool. Rather, it may serve as an adjunctive biomarker that complements established tumor- and liver-related factors. Further studies are needed to determine whether the CALLY index improves discrimination, calibration, and clinical decision-making when added to comprehensive prognostic models.

In this study, propensity-score matching was used as a sensitivity analysis to reduce measured baseline imbalance between patients with high and low CALLY index values. However, the CALLY index is a prognostic biomarker rather than a treatment exposure; therefore, the matched analysis should not be interpreted causally. In addition, propensity-score methods can adjust only for measured covariates, and residual confounding due to unmeasured variables, such as frailty, sarcopenia, antiviral therapy, and detailed treatment after recurrence, cannot be excluded.

This study has several limitations. First, this was a retrospective study from a single institution, which may limit generalizability and introduce selection bias by the exclusion of a substantial proportion of the initially screened cohort. Second, treatment strategies, perioperative management, imaging modalities, and postoperative surveillance may have changed during the study period from 2004 to 2022. The present study included patients treated over a long period from 2004 to 2022. During this period, surgical techniques, perioperative management, imaging modalities, surveillance strategies, antiviral therapy, and treatment options for recurrent HCC changed substantially. To address this issue, we performed a sensitivity analysis stratified by treatment era and found that the association between low CALLY index and worse OS and DFS was consistent in both the 2004–2013 and 2014–2022 cohorts (Supplementary Figure 2). However, this analysis does not fully eliminate residual confounding related to temporal changes, and detailed information on antiviral therapy and post-recurrence treatment was not uniformly available throughout the entire study period. Third, treatment after recurrence was heterogeneous and may have influenced overall survival. Because post-recurrence treatment was determined after the occurrence of recurrence and was influenced by recurrence pattern, liver function, patient condition, and treatment era, it was not included in the primary baseline prognostic model. Therefore, residual confounding related to post-recurrence treatment cannot be fully excluded. Fourth, previous studies have reported different cutoff values for the CALLY index according to cancer type, treatment setting, endpoint, and statistical method, and no universally accepted threshold has been established. Therefore, the cutoff value of 2.2 used in this study was exploratory and requires external validation. To reduce the limitations associated with dichotomization, we additionally analyzed the CALLY index as a continuous variable. Fifth, information regarding the patients’ general condition and cause of death was collected retrospectively from medical records. Consequently, the precise cause of death could not be determined for all cases, and such unmeasured or incompletely measured factors may have influenced postoperative recurrence and survival.

In conclusion, the preoperative CALLY index was independently associated with DFS and OS after hepatic resection for primary HCC. Because it is inexpensive, objective, and readily available from routine laboratory data, the CALLY index may serve as an adjunctive marker for postoperative risk stratification.

## Supporting information

S1 Fig Receiver operating characteristic curve of the CALLY index for survival-group stratification after HCC resection. HCC: hepatocellular carcinoma.
(PDF)

S2 Fig Kaplan–Meier survival curves according to the CALLY index stratified by treatment era. Patients were divided into two cohorts according to the year of surgery: 2004–2013 and 2014–2022. (A) Overall survival in the 2004–2013 cohort. (B) Disease-free survival in the 2004–2013 cohort. (C) Overall survival in the 2014–2022 cohort. (D) Disease-free survival in the 2014–2022 cohort. P values were calculated using the log-rank test. Tick marks indicate censored observations.
(PDF)

S1 Table Missing data by variable. (DOCX)

S2 Table The distribution of initial post-recurrence treatment. (DOCX)

S1 File Dataset. (XLSX)

## Abbreviations

HCC: hepatocellular carcinoma
MELD: model for end-stage liver disease
FIB-4: fibrosis-4
ICG R15: indocyanine green retention rate at 15 min
ALBI: albumin-bilirubin
CRP: C-reactive protein
CALLY: C-reactive protein-albumin-lymphocyte
DFS: disease-free survival
OS: overall survival
EZ-ALBI: easy albumin-bilirubin
AFP: alpha-fetoprotein
DCP: des-γ-carboxy prothrombin
AST: aspartate aminotransferase
HR: hazard ratio
CI: confidence interval
AUC: area under the curve.
