# Supplementary material for: Association between the preoperative C-reactive protein-albumin-lymphocyte index and prognosis after hepatic resection for hepatocellular carcinoma
Source: PLoS One. 2026 Sep 30;21(9):e0358776. doi: 10.1371/journal.pone.0358776 (PMC13626314; doi:10.1371/journal.pone.0358776)

Supplementary Figure 1

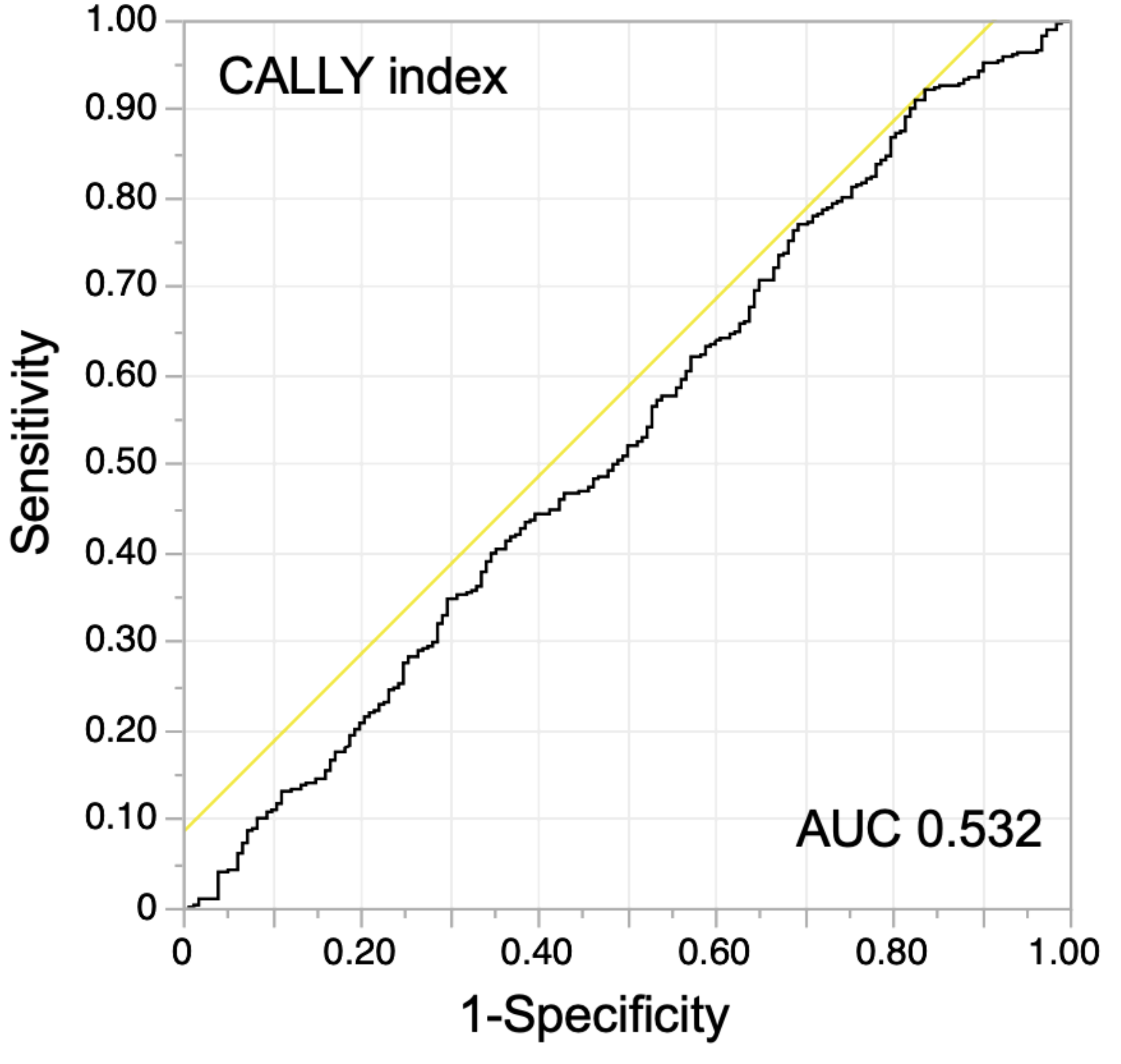

Supplement: Supplementary file 1 [file pone.0358776.s001.pdf]
