# Supplementary material for: Association between the preoperative C-reactive protein-albumin-lymphocyte index and prognosis after hepatic resection for hepatocellular carcinoma
Source: PLoS One. 2026 Sep 30;21(9):e0358776. doi: 10.1371/journal.pone.0358776 (PMC13626314; doi:10.1371/journal.pone.0358776)
Supplement: Supplementary file 2 [file pone.0358776.s002.pdf]

Supplementary Figure 2

A (2004-2013, OS)

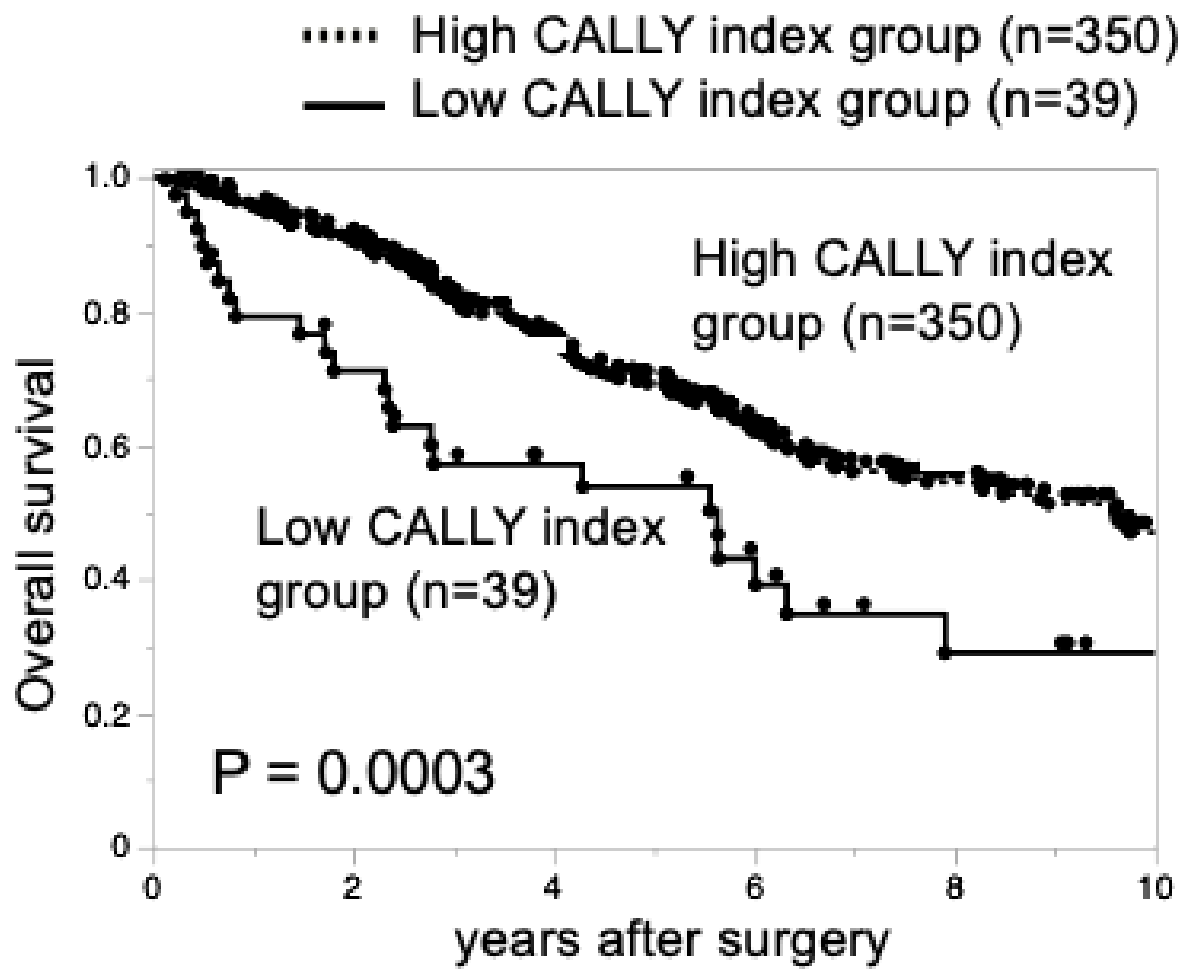

Numbers at risk

Years	0	2	4	6	8	10
High CALLY index	350	301	224	145	97	51
Low CALLY index	39	27	18	12	6	3

C (2014-2022, OS)

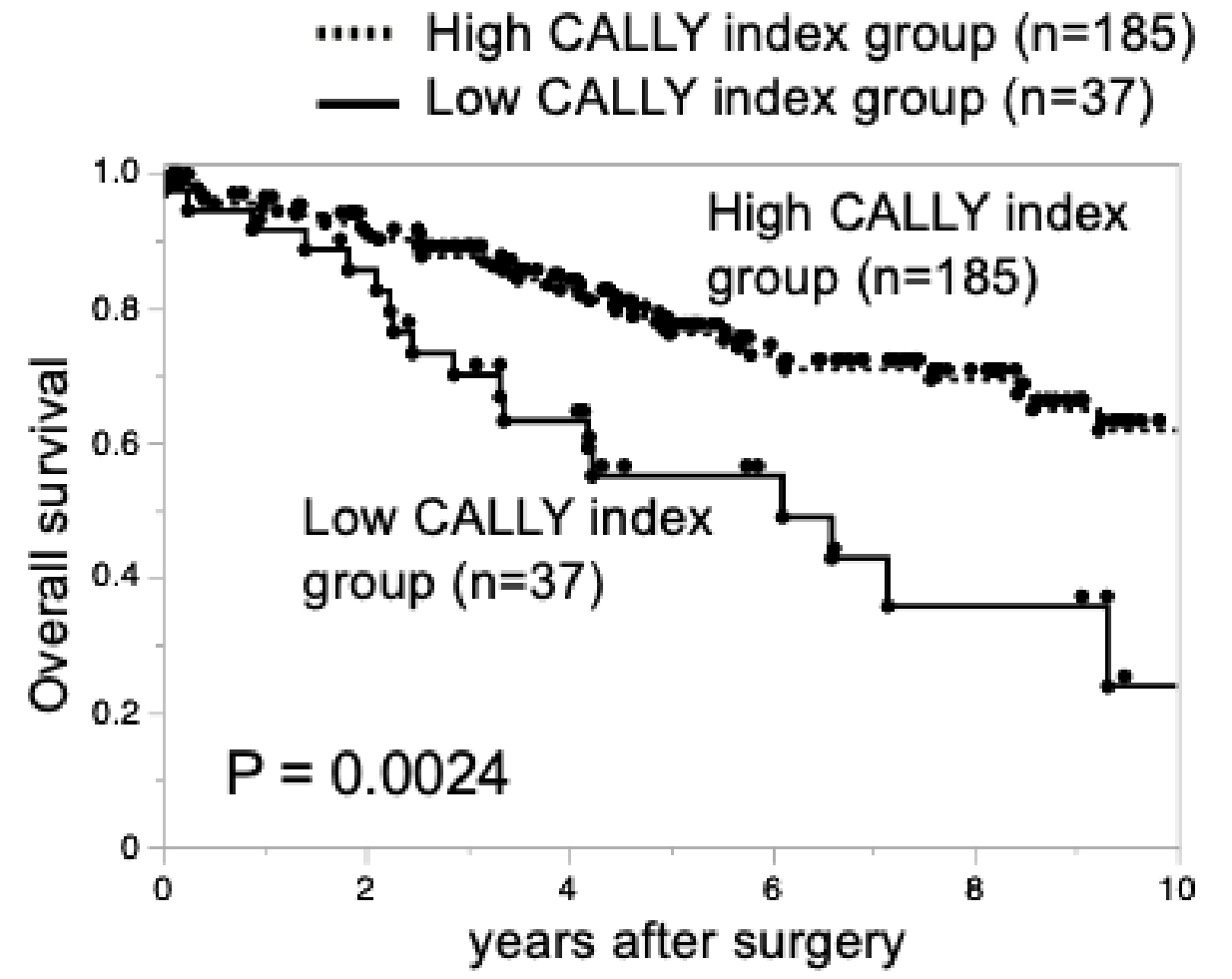

Numbers at risk

Years	0	2	4	6	8	10
High CALLY index	185	150	111	68	41	13
Low CALLY index	37	29	19	10	6	2

B (2004-2013, DFS)

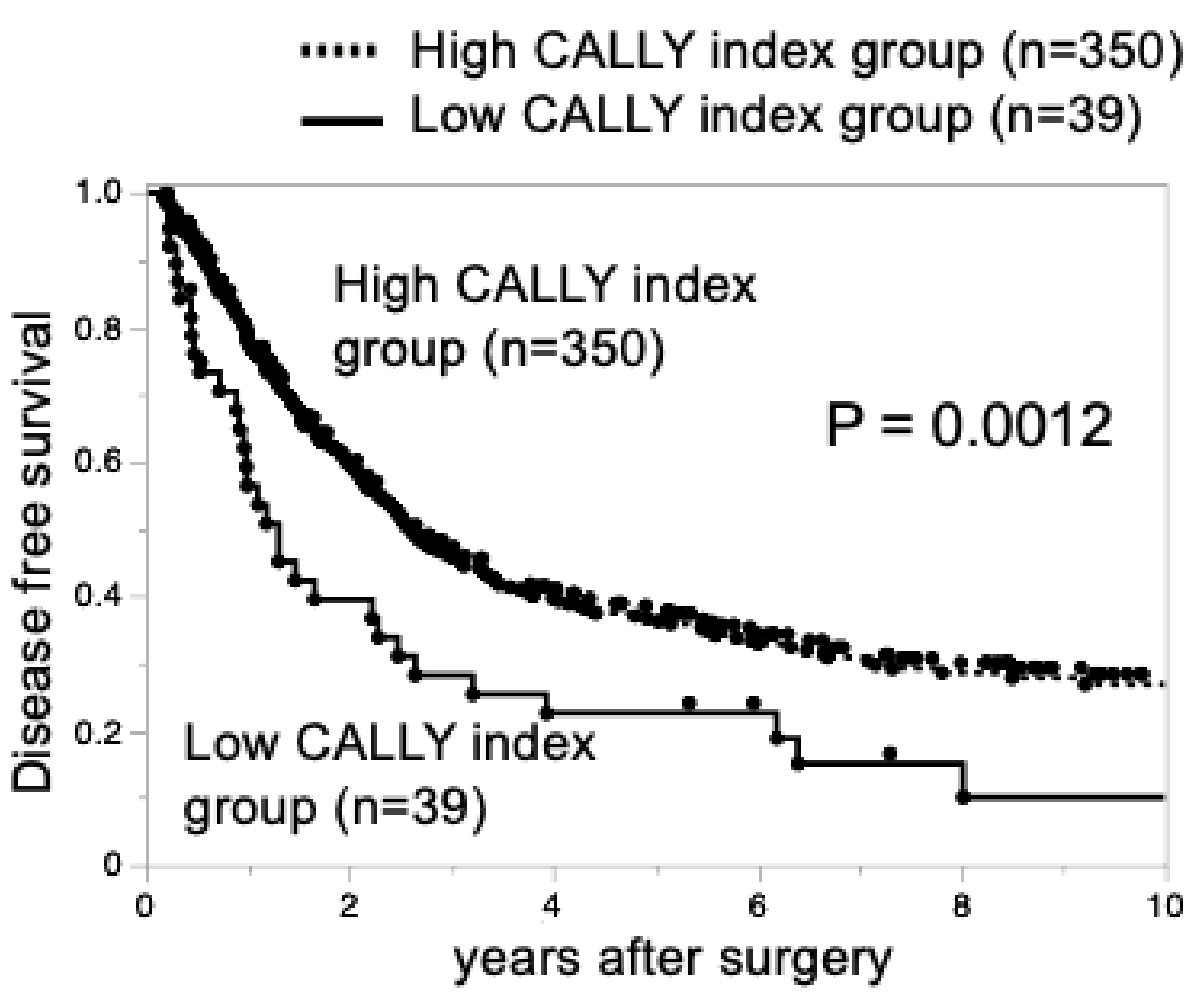

Numbers at risk

Years	0	2	4	6	8	10
High CALLY index	350	190	112	75	46	22
Low CALLY index	39	15	9	7	4	3

D (2014-2022, DFS)

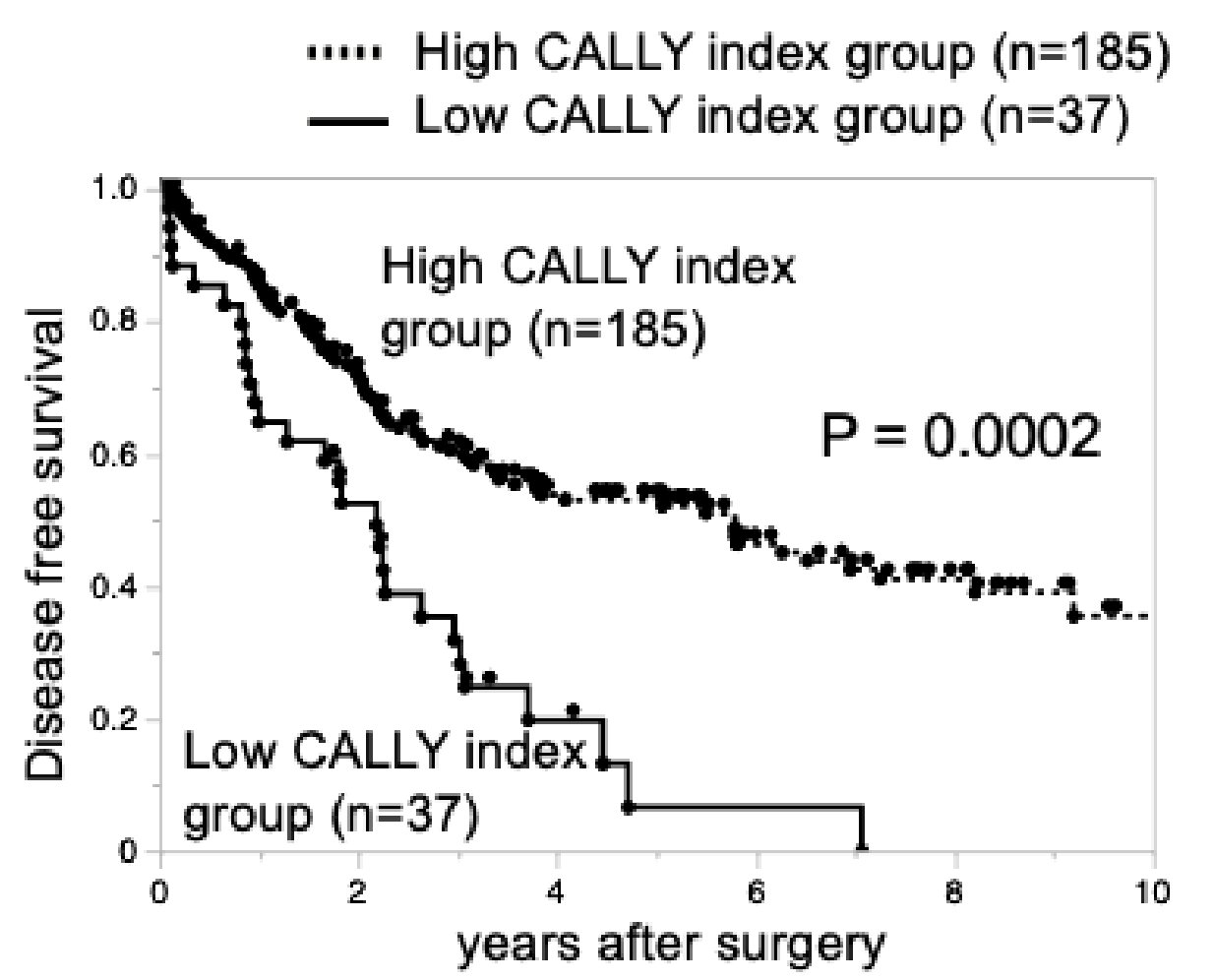

Numbers at risk

Years	0	2	4	6	8	10
High CALLY index	185	115	68	39	24	9
Low CALLY index	37	17	5	2	0	0
