# Supplementary material for: Association between the preoperative C-reactive protein-albumin-lymphocyte index and prognosis after hepatic resection for hepatocellular carcinoma
Source: PLoS One. 2026 Sep 30;21(9):e0358776. doi: 10.1371/journal.pone.0358776 (PMC13626314; doi:10.1371/journal.pone.0358776)
Supplement: Supplementary file 3 [file pone.0358776.s003.docx]

**Supplementary Table 1.** Missing data by variable

| Variable | Available, n | Missing, n |
| --- | --- | --- |
| Diabetes mellitus | 610 | 1 |
| Albumin | 611 | 0 |
| CRP | 611 | 0 |
| Lymphocyte count | 611 | 0 |
| AFP | 611 | 0 |
| DCP | 611 | 0 |
| Tumor size | 611 | 0 |
| Vascular invasion | 611 | 0 |
| Survival outcome | 611 | 0 |

AFP, alpha-fetoprotein; DCP, des-γ-carboxy prothrombin
