# Supplementary material for: Association between the preoperative C-reactive protein-albumin-lymphocyte index and prognosis after hepatic resection for hepatocellular carcinoma
Source: PLoS One. 2026 Sep 30;21(9):e0358776. doi: 10.1371/journal.pone.0358776 (PMC13626314; doi:10.1371/journal.pone.0358776)
Supplement: Supplementary file 4 [file pone.0358776.s004.docx]

**Supplementary Table 2.** The distribution of initial post-recurrence treatment

| Treatment after recurrence | High CALLY index group | Low CALLY index group |
| --- | --- | --- |
| Repeat hepatic resection | 51.3(%) | 43.7(%) |
| Radiofrequency ablation | 9.8(%) | 12.2(%) |
| TACE | 28.8(%) | 34.3(%) |
| Systemic therapy | 2.9(%) | 4.9(%) |
| Radiotherapy | 4.3(%) | 2.4(%) |
| Best supportive care | 2.5(%) | 0(%) |
| Unknown/ not available | 0.3(%) | 2.4(%) |

CALLY, C-reactive protein-albumin-lymphocyte; TACE, transcatheter arterial chemoembolization.
