# Supplementary material for: Association between the preoperative C-reactive protein-albumin-lymphocyte index and prognosis after hepatic resection for hepatocellular carcinoma
Source: PLoS One. 2026 Sep 30;21(9):e0358776. doi: 10.1371/journal.pone.0358776 (PMC13626314; doi:10.1371/journal.pone.0358776)
Supplement: Supplementary file 5 [file pone.0358776.s006.docx]

**Answers to the Reviewer #1:**

We are grateful for the reviewer’s thoughtful comments and suggestions. Our responses to them are as follows. We changed the manuscript accordingly.

**Q1.**

Excluding 44% of the original cohort may increase the risk of bias.

**Our response to Q1:**

We thank the reviewer for this important comment. We agree that the exclusion of a substantial proportion of the initially screened cohort may introduce selection bias and reduce the generalizability of the findings. In the revised manuscript, we have expanded the study flow diagram to provide the detailed reasons for exclusion from the original cohort of 1,089 patients. Specifically, patients were excluded because of non-HCC liver tumors, recurrent HCC, and missing laboratory data required for calculation of the CALLY index. Furthermore, we have explicitly acknowledged this issue as a limitation in the Discussion.

The Methods, Figure 1, and Discussion sections have been revised accordingly.

**Q2.**

There is a marked imbalance between the two groups, which may reduce the precision of the results.

**Our response to Q2:**

We agree with the reviewer. In the original manuscript, substantial baseline imbalances were observed between the high and low CALLY index groups. To address this concern, we revised the propensity-score analysis by incorporating clinically relevant covariates associated with prognosis after HCC resection, including age, sex, viral etiology, liver functional reserve, tumor markers, tumor burden, and pathological factors, as appropriate (Table 5).

The above is described in the results section and in Table 5.

**Q3.**

What were the postoperative interventions, like TACE, RF, or chemotherapy, because all of these might affect the outcome.

**Our response to Q3:**

Thank you for this important comment. We agree that treatment after recurrence may influence postoperative outcomes, particularly overall survival. In the revised manuscript, we added information regarding the initial treatment administered for the first recurrence, including repeat hepatic resection, radiofrequency ablation, transarterial chemoembolization/transarterial embolization, systemic therapy, radiotherapy, best supportive care, and unknown or unavailable treatment information.

Because post-recurrence treatment was selected according to recurrence pattern, residual liver function, patient condition, treatment era, and physician judgment, the treatment strategies were heterogeneous. In addition, these treatments were post-baseline variables that occurred after surgery and after recurrence, and therefore were not included in the primary Cox regression model evaluating the prognostic association of the preoperative CALLY index. However, we agree that post-recurrence treatment may have affected overall survival. We have therefore added the treatment distribution as Supplementary table 2 and acknowledged this issue as a limitation in the Discussion.

**Q4.**

What was the cause of death, recurrence, liver failure, or any other cause?

**Our response to Q4:**

Thank you for this important comment. We agree that the cause of death is important for interpreting overall survival. We attempted to review the causes of death from the available medical records. However, because this was a retrospective study including patients treated over a long period, detailed cause-of-death information was not available for all deceased patients. Therefore, we could not perform a reliable cause-specific survival analysis. We have added this point as a limitation in the revised Discussion.

**Q5.**

Potential residual confounding despite propensity-score matching, as important variables such as frailty, sarcopenia, antiviral therapy, and performance status were not included.

**Our response to Q5:**

Thank you for this important comment. We agree that residual confounding may remain despite propensity-score matching, particularly because host-related factors such as frailty, sarcopenia, antiviral therapy, and performance status may influence long-term outcomes after hepatic resection for HCC.

In response to this comment, we reviewed the available clinical data and added performance status to the baseline variables. Performance status has now been included in Table 5 to better characterize the matched cohort. In addition, where applicable, performance status was incorporated into the revised adjustment strategy.

However, frailty, sarcopenia, and antiviral therapy were not uniformly available for all patients throughout the study period, particularly because this retrospective cohort included patients treated over a long period from 2004 to 2022. Therefore, these variables could not be reliably incorporated into the propensity-score model or multivariable analyses. We have acknowledged this issue more explicitly in the revised Discussion as a limitation.

**Answers to the Reviewer #2:**

We appreciate your thoughtful comments and suggestions. Our responses to the comments are as follows.

**Major comments**

**Q1.**
The manuscript should not currently be described as a validation study.
The CALLY cutoff of 2.2 appears to have been selected using ROC analysis in the same cohort in which its prognostic performance was subsequently evaluated. This represents cutoff derivation and apparent performance assessment rather than independent validation. The authors should clarify whether 2.2 was prespecified from a previous study or derived from the present data. If it was derived from this cohort, the title and conclusions should be revised, or appropriate internal validation using bootstrap resampling or cross-validation should be performed. Ideally, a previously published cutoff should be evaluated without data-driven modification.

**Our response to Q1:**

Thank you for this important comment. We agree with the reviewer that the original wording may have inappropriately implied that the present study was an independent validation study. The cutoff value of 2.2 was derived from the present cohort using ROC analysis and was not prespecified from an independent external cohort. Therefore, this analysis represents exploratory cutoff derivation and apparent performance assessment rather than independent external validation.

In response to this comment, we have removed the term “validation study” from the title and manuscript. We have also revised the Methods section to clarify that the cutoff value of 2.2 was derived from the present dataset and should be interpreted as exploratory. To reduce reliance on dichotomization, we additionally analyzed the CALLY index as a continuous variable in Cox proportional hazards models.

The Results and Discussion sections have been revised accordingly. We have moderated the conclusions and now state that the preoperative CALLY index was independently associated with DFS and OS, rather than claiming that this study provides definitive validation of the CALLY index as a prognostic biomarker. We have also added that further external validation is required to determine the optimal cutoff value and confirm clinical utility.

**Q2.**

The cutoff-selection procedure is insufficiently described.
The authors state that ROC analysis was used for “survival-group stratification,” but they do not specify the outcome, time horizon, censoring method, or criterion used to select the cutoff. It is unclear whether the cutoff was based on overall survival, disease-free survival, early recurrence, the Youden index, or another criterion. These details must be reported. The supplementary ROC figure should also be included and accessible to reviewers.

The authors should additionally analyze the CALLY index as a continuous variable rather than relying exclusively on dichotomization. Restricted cubic splines would help assess whether the association is linear and whether 2.2 represents a clinically meaningful threshold.

**Our response to Q2:**

Thank you for this important comment. We agree that the cutoff-selection procedure was insufficiently described in the original manuscript. In the revised manuscript, we have clarified that the cutoff value of 2.2 for the CALLY index was derived from receiver operating characteristic curve analysis using overall survival as the outcome. The optimal cutoff was selected according to the Youden index. We have also ensured that the corresponding ROC curve is included as Supplementary Figure 1 and is accessible to reviewers.

Because this cutoff value was derived from the present cohort and was not prespecified from an external dataset, we now describe it as an exploratory cutoff. To reduce reliance on dichotomization, we additionally analyzed the CALLY index as a continuous variable in Cox proportional hazards models. The Methods and Discussion sections have been revised accordingly, and the limitations associated with cohort-derived cutoff selection have been explicitly acknowledged.

**Q3.**

Standard ROC analysis is not adequate for censored survival outcomes.
Overall survival and disease-free survival are time-to-event endpoints with censoring. The reported AUC values for “predicting DFS” and “predicting OS” have no specified time horizon, and it is unclear whether censoring was handled. The authors should use time-dependent ROC analysis at prespecified time points, such as 1, 3, and 5 years, or report Harrell’s C-index. Confidence intervals should be provided for all discrimination measures.

Comparisons between the CALLY index and ALBI, EZ-ALBI, FIB-4, MELD, and Child-Pugh should include confidence intervals and formal statistical comparisons. Simply identifying the numerically highest AUC does not establish superior prognostic performance.

**Our response to Q3:**

Thank you for this important methodological comment. We agree that conventional ROC analysis is not appropriate as the primary method for evaluating censored time-to-event outcomes such as DFS and OS. In the revised manuscript, we therefore performed time-dependent ROC analyses using EZR at prespecified time points of 1, 3, and 5 years after hepatic resection. Time-dependent AUCs were calculated for the CALLY index and established liver function indices, including ALBI score, EZ-ALBI score, FIB-4 index, MELD score, and Child-Pugh classification. The revised Table 4A now presents the time-dependent AUCs. We also revised the terminology throughout the manuscript by replacing “diagnostic accuracy” with “prognostic discrimination” when referring to DFS and OS.

We agree that the discrimination of the CALLY index was modest. Therefore, we have moderated the interpretation of the results and no longer state that the CALLY index has superior diagnostic accuracy. Instead, we state that the CALLY index showed modest prognostic discrimination and may provide adjunctive prognostic information. Conventional ROC analysis was retained only for early recurrence within 1 year after surgery, which was evaluated as a binary outcome.

**Q4.**

The discrimination of the CALLY index is modest and should not be overstated.
The reported AUC values are approximately 0.60 for DFS and early recurrence. This represents limited discrimination. Statements concerning “highest diagnostic accuracy,” clinical usefulness, and added prognostic value should therefore be moderated. The term “prognostic discrimination” is more appropriate than “diagnostic accuracy.”

To support claims of added value, the authors should compare a baseline clinicopathological model with the same model plus the CALLY index. Changes in the C-index, model likelihood, calibration, and, if clinical implementation is claimed, decision-curve analysis should be considered.

**Our response to Q4:**

Thank you for this important comment. We agree that the prognostic discrimination of the CALLY index was modest and that its clinical utility should not be overstated. As described in our response to Major Comment 3, we performed time-dependent ROC analyses for DFS and OS and revised the terminology by replacing “diagnostic accuracy” with “prognostic discrimination.” We have also removed statements suggesting that the CALLY index has superior or high predictive performance.

In the revised manuscript, we now state that the CALLY index showed modest prognostic discrimination and may provide adjunctive prognostic information rather than serving as a stand-alone prognostic tool. We have also revised the Results, Discussion, and Conclusions to avoid overinterpretation of the AUC values.

We agree that formal assessment of incremental clinical value would require comparison of a baseline clinicopathological model with and without the CALLY index, as well as evaluation of calibration and decision-curve analysis. These analyses were not performed in the present study. Therefore, we have avoided claiming that the CALLY index improves clinical decision-making and have added this point as a limitation. We now state that further studies are required to determine whether the CALLY index provides incremental value beyond established tumor- and liver-related prognostic factors.

**Q5.**

The Cox regression strategy requires substantial clarification and revision.
The numbers of recurrence events and deaths are not reported. These numbers are necessary to assess the number of events per model parameter and the risk of model overfitting. The authors should report:

Number of DFS events, recurrences, deaths, and censored observations.
Median follow-up calculated using an appropriate method, preferably reverse Kaplan-Meier.
Proportional-hazards assumption testing for each Cox model.
The complete prespecified covariate-selection strategy.
Missing-data handling and the number of patients included in each model.

Several continuous variables were dichotomized using cohort-derived means or thresholds, including age, AFP, DCP, tumor size, AST, ALT, and ICG R15. This causes loss of information and may produce unstable or biased estimates. These predictors should preferably be analyzed continuously, with transformations or spline functions where appropriate.

Potential collinearity must also be addressed because the CALLY index contains albumin, CRP, and lymphocyte count, while other models include albumin-related measures such as ALBI or EZ-ALBI.

**Our response to Q5:**

Thank you for these important methodological comments. We agree that the Cox regression strategy required further clarification. In the revised manuscript, we have added the numbers of DFS events, recurrences, deaths, and censored observations. We also added the median follow-up period and clarified the number of patients included in the analyses.

We have revised the Statistical Analysis section to describe the covariate-selection strategy for the Cox proportional hazards models. Variables were selected based on clinical relevance and statistical significance in univariate analysis, while taking into account the number of observed events to reduce the risk of overfitting. We also assessed the proportional hazards assumption using graphical and residual diagnostics available in JMP and clarified this in the revised Methods section.

We agree that dichotomization of continuous variables may lead to loss of information and unstable estimates. Therefore, in response to this comment, we additionally analyzed the CALLY index as a continuous variable. The continuous-variable analysis showed that the CALLY index remained significantly associated with postoperative outcomes, supporting the robustness of the association without relying exclusively on the cutoff value. We have added these results to the revised Results section and tables.

We also addressed potential collinearity. Because the CALLY index includes albumin, CRP, and lymphocyte count, these individual components were not included simultaneously with the CALLY index in the same multivariable model. In addition, albumin-based indices such as ALBI and EZ-ALBI were interpreted cautiously when considered together with the CALLY index. We have added this explanation to the Methods and Discussion sections.

Finally, we clarified the handling of missing data. Complete-case analysis was performed for variables included in each model, and the number of patients included in each analysis is now reported. We have also acknowledged residual confounding, possible overfitting, and information loss due to dichotomization as limitations.

**Q6.**

The propensity-score matching analysis is inadequately reported.
The manuscript should specify the exact propensity-score model, all included covariates, matching ratio, matching algorithm, whether matching was performed with or without replacement, whether the caliper was applied to the propensity score or its logit, and how common support and missing values were handled. “Caliber” should be corrected to “caliper.”

Balance should not be assessed using nonsignificant P values. Standardized mean differences before and after matching should be reported, preferably with a balance or Love plot. A standardized mean difference below a prespecified threshold should be used to assess balance.

Because the observations are matched, the survival analyses should account for matched pairs, for example through a stratified Cox model or cluster-robust standard errors. The authors should report matched hazard ratios with 95% confidence intervals, not only log-rank P values.

The authors should also clarify the estimand and purpose of propensity-score matching. The CALLY index is a prognostic biomarker rather than a treatment exposure, and causal terms such as “effect” or “impact” should be avoided.

**Our response to Q6:**

Thank you for this important comment. We agree that the propensity-score matching analysis was inadequately described in the original manuscript and that balance should not be assessed using nonsignificant P values alone.

In the revised manuscript, we have substantially expanded the description of the propensity-score matching procedure. The propensity score was estimated using a logistic regression model with the CALLY index group as the dependent variable. One-to-one nearest-neighbor matching was performed without replacement using a caliper width of 0.2 of the standard deviation of the logit of the propensity score. Patients outside the region of common support were not matched, and complete-case analysis was performed for variables included in the propensity-score model.

We also clarified that covariate balance was assessed using standardized mean differences, and an absolute standardized mean difference <0.1 was considered to indicate adequate balance.

Regarding survival analysis in the matched cohort, we revised the text to clarify that survival outcomes after matching were evaluated using Kaplan–Meier curves and log-rank tests. We did not estimate matched hazard ratios because the propensity-score matched analysis was performed as a sensitivity analysis to examine whether the survival separation between the high and low CALLY index groups persisted after reducing measured baseline imbalance, rather than as a primary model for effect estimation.

Finally, we clarified that the CALLY index is a prognostic biomarker rather than a treatment exposure; therefore, the propensity-score matched analysis was interpreted as a sensitivity analysis of prognostic association, not as a causal effect analysis. We have avoided causal terms such as “effect” and “impact” throughout the revised manuscript.

**Q7.**

Important clinical confounders and temporal changes need consideration.
The study covers the period from 2004 to 2022, during which surgical techniques, antiviral treatment, imaging, surveillance, and management of recurrent HCC changed substantially. Year of surgery or treatment era should be examined in sensitivity analyses.

The authors should clarify whether established prognostic variables were available, including tumor stage, cirrhosis, portal hypertension, performance status, resection extent, surgical margin, antiviral treatment, and treatment after recurrence. The absence of these variables may cause residual confounding.

**Our response to Q7:**

Thank you for this important comment. We agree that temporal changes during the long study period may have influenced postoperative outcomes. The present cohort included patients who underwent hepatic resection between 2004 and 2022, during which surgical techniques, perioperative management, imaging modalities, surveillance strategies, antiviral treatment, and treatment options for recurrent HCC changed substantially.

In response to this comment, we performed an additional sensitivity analysis according to treatment era. Patients were divided into two cohorts based on the year of surgery: 2004–2013 and 2014–2022. Within each treatment era, OS and DFS were compared between the high and low CALLY index groups using Kaplan–Meier curves and log-rank tests. In both treatment eras, the low CALLY index group showed significantly worse OS and DFS than the high CALLY index group. These results have been added as Supplementary Figure 2.

We also reviewed the availability of clinically relevant variables, including performance status, resection-related factors, and post-recurrence treatment, and added available information to the revised manuscript where possible. However, some variables, such as detailed antiviral therapy, frailty, sarcopenia, portal hypertension, and detailed post-recurrence treatment, were not uniformly available throughout the entire study period. Therefore, we have acknowledged residual confounding related to temporal changes and unavailable clinical variables as a limitation in the revised Discussion.

**Q8.**

Laboratory measurement and calculation of the CALLY index require clarification.
The authors should report:

The permitted interval between blood collection and surgery.
Whether patients with active infection or acute inflammatory disorders were excluded.
The CRP assay and lower detection limit.
How CRP values of zero or below the detection limit were handled, because CRP appears in the denominator.
The units used for CRP and lymphocyte count.
The distribution of lymphocyte count and the CALLY index in the total cohort and both groups.

The definitions “high >2.2” and “low <2.2” leave patients with a value exactly equal to 2.2 unclassified. One group should use an inclusive boundary.

**Our response to Q8:**

Thank you for this important comment. We agree that the laboratory measurement conditions and calculation of the CALLY index required clarification, particularly because CRP is included in the denominator of the formula.

In the revised manuscript, we have added detailed information regarding laboratory measurements. Preoperative laboratory data were obtained within 7 days before hepatic resection. Serum albumin was expressed in g/dL, lymphocyte count in cells/μL, and CRP in mg/dL. The lower reporting limit for CRP at our institution was 0.01 mg/dL, and no patient had a CRP value of zero. For patients with CRP values reported as zero or below the lower detection limit, no such patients were present. This handling has now been explicitly described in the Methods section.

We also reviewed the medical records for clinically apparent active infection or acute inflammatory disease at the time of preoperative blood sampling. Patients with such conditions were excluded. This point has been added to the Methods sections.

Finally, we corrected the group definition to avoid ambiguity at the cutoff value. Patients were classified into the high CALLY index group (≥2.2) and the low CALLY index group (<2.2).

**Q9.**

Patient selection and missing data should be reported transparently.
The flow diagram shows a reduction from 1,089 patients undergoing hepatic resection to 611 included patients, but it does not provide the number excluded for each reason. The authors should report separate counts for non-HCC tumors, recurrent HCC, missing laboratory data, and insufficient follow-up.

Missingness is already apparent in Table 1, where diabetes mellitus totals 610 rather than 611 patients. All missing values should be reported by variable, and the handling of missing data should be described. Complete-case analysis should be justified or multiple imputation considered where appropriate.

**Our response to Q9:**

Thank you for this important comment. We agree that the patient selection process and handling of missing data were insufficiently described in the original manuscript.

In the revised manuscript, we have revised Figure 1 to provide a detailed flow diagram of patient selection. Of the 1,089 patients who underwent hepatic resection for liver tumors during the study period, 478 were excluded: 68 had non-HCC liver tumors, 381 had recurrent HCC, and 29 had missing laboratory data required for calculation of the CALLY index. Finally, 611 patients who underwent initial hepatic resection for primary HCC were included in the analysis.

We also reviewed missing data for the variables used in the analyses and added Supplementary Table 1, which summarizes the number of available and missing values for each variable. Diabetes mellitus status was missing in one patient, which explains why the total number for this variable was 610 rather than 611 in Table 1. In contrast, data for albumin, CRP, lymphocyte count, AFP, DCP, tumor size, vascular invasion, and survival outcomes were available for all 611 patients.

We have clarified in the Statistical Analysis section that complete-case analysis was performed for variables with missing data. Because missing data were minimal, multiple imputation was not performed. We have also added this point to the revised manuscript and acknowledged the possibility of selection bias related to patient exclusion as a limitation.

**Q10.**

Descriptive statistics and table reporting need revision.
Mean ± standard error is generally not appropriate for describing patient-level baseline distributions. Normally distributed variables should be reported as mean ± standard deviation, while skewed variables should be reported as median and interquartile range. Several variables, including CRP, blood loss, tumor size, and operative duration, may be skewed and require assessment.

Please verify the value “total bilirubin 0.81 ± 1.1 mg/dL” in Table 5, which appears to be a typographical or reporting error. CRP units should also be added to Table 1. The DCP distributions and corresponding P value should be rechecked due to the markedly asymmetric values.

**Our response to Q10:**

Thank you for this important comment. We agree that mean ± standard error is not appropriate for describing patient-level baseline distributions and may imply excessive precision. In response to the reviewer’s comment, we revised Tables 1 and 5 and now present all continuous variables as median [interquartile range]. Categorical variables are presented as numbers and percentages.

Accordingly, between-group comparisons for continuous variables were performed using the Mann–Whitney U test. The Statistical Analysis section and the footnotes of Tables 1 and 5 have been revised to clarify the presentation of variables and the statistical tests used.

We also carefully rechecked the values in the tables. The value of total bilirubin in Table 5 was confirmed to be a typographical/reporting error and has been corrected. The unit of CRP has been added to Table 1 as mg/dL. In addition, because DCP showed a skewed distribution, we rechecked the DCP distributions and the corresponding P value using the Mann–Whitney U test.

**Q11.**

The data-availability statement is incomplete.
The statement that all data are available without restriction does not indicate where the data can be accessed. The authors should provide a repository name, permanent DOI or accession link, and a de-identified minimal dataset sufficient to reproduce the tables, survival analyses, and propensity-score analysis. Statistical code should also be shared where possible.

**Our response to Q11:**

Thank you for this important comment. We agree that the original data-availability statement was incomplete because it did not specify where the data could be accessed.

In the revised submission, we have added a de-identified minimal dataset as a Supporting Information file. This dataset includes the variables necessary to reproduce the baseline tables, survival analyses, time-dependent ROC analyses, and propensity-score matching analysis. We have also revised the Data Availability Statement to clearly indicate that the relevant data underlying the findings are available in the Supporting Information files. Statistical analyses were performed using JMP Pro and EZR as described in the Methods section.

The revised Data Availability Statement now reads as follows:
“All relevant data underlying the findings of this study are provided in the Supporting Information files. The de-identified minimal dataset used to reproduce the baseline tables, survival analyses, time-dependent ROC analyses, and propensity-score matching analysis is provided as S1 Dataset.”

**Q12.**

Ethics and consent reporting should be clarified.
The authors should specify whether informed consent was written, oral, or obtained through an opt-out procedure. Given the retrospective inclusion of patients treated from 2004 onward, the procedure for deceased or unavailable patients should be explained. The manuscript should also clarify whether the research dataset was anonymized and whether investigators had access to identifiable information.

**Our response to Q12:**

Thank you for this important comment. We agree that the ethics and consent procedures required clarification.

In the revised manuscript, we have clarified that this retrospective observational study was approved by the Ethics and Indications Committee of Hiroshima Red Cross Hospital and Atomic-bomb Survivors Hospital (approval No. 2021-029). Because of the retrospective nature of the study, the requirement for written informed consent was waived by the ethics committee, and an opt-out approach was used. Information regarding the study was disclosed, and patients or their representatives were given the opportunity to decline participation. This opt-out procedure also applied to patients who had died or could not be contacted.

We have also clarified the handling of patient data. Authorized investigators accessed clinical records only for data collection. After data extraction, direct personal identifiers were removed, and statistical analyses were performed using a de-identified dataset. This information has been added to the revised Ethics Statement.

**Minor comments**

**Q1.**

The abstract should report the principal adjusted hazard ratios and 95% confidence intervals rather than only stating statistical significance.

**Our response to Q1:**

Thank you for this suggestion. We have revised the Abstract to include the principal adjusted hazard ratios and 95% confidence intervals for DFS and OS. Specifically, we now report that a low CALLY index was independently associated with worse DFS and OS, with the corresponding HRs, 95% CIs, and P values.

**Q2.**

Replace causal wording such as “impact” and “effect” with “association” unless a causal analysis is justified.

**Our response to Q2:**

Thank you for this comment. We agree that causal wording is not appropriate for this retrospective observational study. We have revised the manuscript throughout by replacing causal terms such as “impact” and “effect” with non-causal terms such as “association,” “prognostic association,” and “adjunctive prognostic information.”

**Q3.**

Figure 1 should present all exclusion categories and numbers.

**Our response to Q3:**

Thank you for this comment. We have revised Figure 1 to show all exclusion categories and the corresponding numbers. Of the 1,089 initially screened patients, 68 were excluded because of non-HCC liver tumors, 381 because of recurrent HCC, and 29 because of missing laboratory data required for calculation of the CALLY index. The final study cohort consisted of 611 patients with primary HCC.

**Q4.**

Figure legends should state the statistical test used and explain the censoring marks.

**Our response to Q4:**

Thank you for this helpful suggestion. We have revised the figure legends to state that P values for Kaplan–Meier survival curves were calculated using the log-rank test. We have also added an explanation that tick marks indicate censored observations.

**Q5.**

The manuscript is generally intelligible, but minor grammatical and terminology corrections are required throughout.

**Our response to Q5:**

Thank you for this comment. We have carefully reviewed the manuscript and corrected grammatical, stylistic, and terminology issues throughout. We also standardized key terms, including “preoperative CALLY index,” “propensity-score matching,” “caliper,” and “prognostic discrimination.”

**Answers to the Reviewer #3:**

We are grateful for the reviewer’s thoughtful comments and suggestions. Our responses to them are as follows. We changed the manuscript accordingly.

**Q1.**

High and Low CALLY group is classified as value ><2.2. What is the rational for considering 2.2 as demarcating value?

**Our response to Q1:**

Thank you for this important comment. We agree that the rationale for using 2.2 as the cutoff value for the CALLY index was insufficiently described in the original manuscript. To our knowledge, no universally accepted cutoff value for the CALLY index has been established across cancer types or even among patients with HCC. Previous studies have used different cutoff values depending on the cancer type, treatment setting, endpoint, and statistical method. Therefore, the cutoff value of 2.2 in the present study should not be interpreted as an externally validated universal threshold.

In the original analysis, the cutoff value of 2.2 was derived from the present cohort using ROC analysis for survival-group stratification. We have now clarified this point in the Methods section. In response to the reviewer’s comment, we also performed additional analyses treating the CALLY index as a continuous variable to avoid relying exclusively on dichotomization. Furthermore, we have revised the wording in the Results and Discussion to state that the cutoff-based analysis was exploratory and that the optimal cutoff value requires further validation in independent cohorts.

**Q2.**

Line 170. check the spelling-"caliper"

**Our response to Q2:**

Thank you for pointing that out. We have corrected the spelling error.

**Q3.**

Conclusion in abstract: The perioperative CALLY Index is an independent prognostic factor after hepatic resection for primary HCC. Conclusion in Full Text: The perioperative CALLY Index is independently associated with DFS and OS after primary HCC resection. Could this study alone establish CALLY Index as independent prognostic factor??

**Our response to Q3:**

Thank you for this important comment. We agree that the original wording of the conclusion may have overstated the findings of this retrospective single-center study. The CALLY index was calculated using preoperative laboratory data; therefore, we have revised “perioperative CALLY index” to “preoperative CALLY index” throughout the manuscript for consistency.

We also agree that this study alone cannot definitively establish the CALLY index as a validated prognostic factor. Therefore, we have revised the Abstract Conclusion and the full-text Conclusion to state that the preoperative CALLY index was “independently associated with DFS and OS” rather than definitively describing it as an “independent prognostic factor.”
